# Dietary Patterns and Prostate Cancer: CAPLIFE Study

**DOI:** 10.3390/cancers14143475

**Published:** 2022-07-17

**Authors:** Macarena Lozano-Lorca, Margarita Rodríguez-González, Inmaculada Salcedo-Bellido, Fernando Vázquez-Alonso, Miguel Arrabal, Benita Martín-Castaño, María-José Sánchez, José-Juan Jiménez-Moleón, Rocío Olmedo-Requena

**Affiliations:** 1 Departamento de Enfermería, Facultad de Ciencias de la Salud de Ceuta, Universidad de Granada, 51001 Ceuta, Spain; macarenalozano@ugr.es; 2 Instituto de Investigación Biosanitaria ibs. GRANADA, 18014 Granada, Spain; mariajose.sanchez.easp@juntadeandalucia.es (M.-J.S.); jjmoleon@ugr.es (J.-J.J.-M.); rocioolmedo@ugr.es (R.O.-R.); 3 Departamento de Medicina Preventiva y Salud Publica, Universidad de Granada, 18016 Granada, Spain; margarg98@correo.ugr.es; 4 Consortium for Biomedical Research in Epidemiology and Public Health (CIBERESP), 28029 Madrid, Spain; 5 Urology Department, Virgen de las Nieves University Hospital, 18014 Granada, Spain; fvazquezalonso@gmail.com; 6 Urology Department, Clínico San Cecilio University Hospital, 18016 Granada, Spain; arrabalm8@gmail.com; 7 Las Gabias Primary Health Care Centre, Distrito Sanitario Granada-Metropolitano, 18110 Granada, Spain; benita.martin.sspa@juntadeandalucia.es; 8 Andalusian School of Public Health (EASP), 18011 Granada, Spain

**Keywords:** dietary patterns, prostate cancer, CAPLIFE study, case-control study, principal component analysis

## Abstract

**Simple Summary:**

Although some nutrients, foods, and food groups have been linked to prostate cancer (PCa), their relationship remains unclear. In this sense, dietary patterns allow a global approach to diet. This study evaluated the role of dietary patterns on PCa by tumor aggressiveness and extension. A total of 428 incident PCa cases and 393 controls were included. When comparing scores in the highest vs. lowest tertiles, an unhealthy dietary pattern was associated with higher odds of PCa. This association was observed only for PCa ISUP 1 or 2 tumors and localized PCa cases. We were unable to establish a clear association between Western or Mediterranean dietary patterns and PCa. These results increase the evidence of a possible relationship between diet and PCa. Therefore, future recommendations should focus on avoiding unhealthy dietary patterns.

**Abstract:**

The etiology of prostate cancer (PCa) remains uncertain, and the role of diet is unclear. We aimed to evaluate the role of diet, through dietary patterns, on PCa, considering tumor aggressiveness and extension. The CAPLIFE study is a population-based case-control study including a total of 428 incident PCa cases and 393 controls aged 40–80 years. Dietary information was collected through a validated food frequency questionnaire. Three dietary patterns were identified through principal component analysis: “Mediterranean,” “Western,” and “Unhealthy,” which were categorized into tertiles according to the control group cutoff points. Tumor aggressiveness and extension was determined. Logistic regression models were used to assess the association between dietary patterns and PCa. High adherence to an unhealthy dietary pattern was associated with higher odds of PCa, OR_T3vsT1_ = 1.52 (95% CI 1.02–2.27), especially for cases with ISUP 1–2 and localized PCa tumors. This association was not observed with a Western or Mediterranean pattern. In conclusion, adherence to an unhealthy diet appears to be associated with higher odds of PCa, especially for cases with ISUP 1–2 and localized PCa tumors.

## 1. Introduction

Prostate cancer (PCa) was the second most frequent cancer and the fifth leading cause of cancer death among men in 2020 [1]. Despite its considerable impact, relatively little is known about its etiology. Established risk factors are limited to advancing age, race/ethnicity, family history, and specific genetic polymorphisms, none of which are modifiable. However, the possible implications of other factors such as obesity, physical inactivity, smoking, and diet in the development of PCa remain uncertain [2]. Regarding diet, the Word Cancer Research Foundation and the American Institute for Cancer Research (WCRF/AICR) in their third report from 2018 suggest that there is limited evidence on its association with PCa [3]. In this sense, the WCRF/AICR proposes that high consumption of dairy products and calcium could increase the risk of PCa. On the other hand, the International Agency for Research on Cancer (IARC) classifies only red meat as a possible carcinogen [4].

To date, the role of diet on the risk of developing PCa has been evaluated from different approaches: (i) single nutrients, (ii) foods or food groups, and (iii) dietary patterns. Several studies have addressed diet at the isolated nutrient level, and they suggest that lycopene, selenium, and vitamin E could reduce the risk of PCa [5,6,7]. In contrast, vitamin D, calcium, and zinc are postulated as possible risk factors for PCa [8,9,10]. Following the same line, among the most controversial foods due to their relationship with PCa are milk, red and processed meats, and low intake of fruits and vegetables [11,12,13].

However, the evidence for these approaches is still scarce [14,15]. This could be due to lack of consideration of the complex interactions and synergies that may exist between combinations of different nutrients and foods [16]. Furthermore, it is difficult to analyze the role of individual foods because a typical diet is characterized by a mixture of different foods, where an increase in the consumption of some foods will lead to a decrease in the consumption of others [17]. In this way, the focus of nutritional epidemiology has gradually shifted from single nutrients to dietary patterns [18].

The most important methods for extracting dietary patterns are: (1) a priori or researcher-driven and (2) a posteriori or data-driven approaches. Within the a posteriori approaches, principal component analysis is the most used method [17,19,20]. Using this method, multiple dietary patterns have been identified, such as Western or Traditional, Mediterranean, prudent patterns named according to the foods or food groups consumed [21,22,23,24,25,26,27,28,29]. Thus, adherence to a Western or Traditional pattern has been associated with increased risk of PCa [25,26,27,29], while adherence to a healthy or Mediterranean pattern has been associated with lower risk of PCa [21,28]. In addition, PCa is a heterogeneous disease in terms of aggressiveness, extension, and prognosis, with highly aggressive cases and a high probability of metastases that progress despite interventions, whereas other tumors will never become clinically significant [30,31,32]. Therefore, the role of diet may depend on the aggressiveness or extension of a PCa tumor; however, few studies have considered this in the association between dietary patterns and PCa, producing inconsistent results [21,22,25,26,33,34]. In this sense, to date, only one study has addressed the association between dietary patterns and PCa in a European population, concluding that a Mediterranean pattern could protect against highly aggressive tumors [22]. However, the role played by other patterns such as Western or unhealthy remains uncertain in this population.

Given the increase in the incidence of PCa, that the etiology of this tumor is currently largely unknown and information on modifiable factors is lacking, the identification of multiple patterns whose association with PCa is not clear, and the scarce consideration of tumor aggressiveness and extension in the association between dietary patterns and PCa, this study aimed to evaluate the role of dietary pattern on PCa, considering tumor aggressiveness and extension, within the CAPLIFE study.

## 2. Materials and Methods

### 2.1. Study Design and Participants

The CAPLIFE study is a population-based case-control study that aims to evaluate the association between lifestyle and PCa. It was carried out at the two main hospitals in Granada (Spain): Virgen de las Nieves and Clínico San Cecilio Hospitals and their catchment areas. Participants were recruited between May 2017 and September 2020. Previous studies have extensively described the methodology of the CAPLIFE study [35,36].

The Ethics Committee for Biomedical Research of Andalusia approved the study protocol in March 2017. Participants were fully informed about the study and signed informed consent. Furthermore, the confidentiality of data was secured, removing any personal identifiers from the dataset.

PCa cases and controls must meet the following criteria for selection: (1) age between 40 and 80 years; (2) residence in the coverage area of the reference hospitals for at least 6 months; and (3) new diagnosis of PCa with histopathological confirmation before receiving treatment (only PCa cases) as defined by the *International Classification of Diseases and Related Health Problems 10th Revision* (ICD-10): C61 [37].

**Figure 1:**
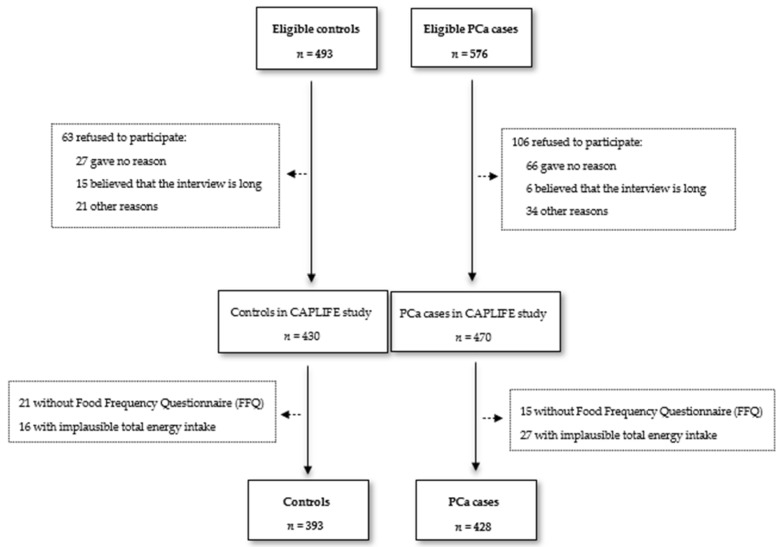
Flowchart CAPLIFE study.

Incident cases were invited to participate from the pathological anatomy lists after confirming that their biopsy had a positive result from the urology services of participating hospitals. Controls were randomly selected from the general population using the lists of general practitioners from primary care centers, and they were frequency-matched by age based on the information obtained from the Granada Cancer Registry. A total of 470 cases and 430 controls were recruited in the CAPLIFE study. Subjects without dietary information or with implausible energy intake (less than 800 kcal/day or more than 4000 kcal/day) were not considered in this analysis [38]. Thus, after applying the exclusion criteria, the resulting sample consisted of 428 cases and 393 controls (Figure 1).

### 2.2. Data Collection

Face-to-face interviews were conducted at baseline by trained interviewers using a structured questionnaire. In this way, sociodemographic data (age, education level, employment, and marital status), first-degree family history of PCa, comorbidities, and lifestyle data (smoking status, physical activity, and sedentary behavior) were collected. In addition, information on self-reported weight and height was obtained for calculating body mass index (BMI).

Specifically, comorbidities were evaluated as: (i) presence of two or fewer comorbidities, and (ii) presence of three or more comorbidities. The comorbidities collected included: diabetes mellitus (type 1 or 2), cardiovascular diseases (stroke, angina pectoris and/or acute myocardial infarction), respiratory diseases (asthma and/or chronic obstructive pulmonary disease), hypertension, mental illnesses (anxiety and/or depression), and other previous cancers. Smoking status was categorized as never smoker (less than 100 cigarettes), former smoker (not having smoked 1 year before), and current smoker (having smoked for at least 6 months and continuing with the habit in the previous year). The International Physical Activity Questionnaire Short Form (IPAQ-SF) was used to collect physical activity and sedentary behavior information. It was validated for the Spanish population and referred to the week before the interview. Three categories were established: low, moderate, and high physical activity [39].

Dietary information for the 12 months before the interview was obtained using a food frequency questionnaire (FFQ), previously validated for the Spanish population, including regional products [40,41]. It collected information on 134 food items, as well as the consumption of beverages. Portion sizes were specified for each item, and 9 consumption frequency options were provided, from “never” to “more than 6 times a day”. Information about total energy intake and intake of both macronutrients and micronutrients was derived from Spanish food composition tables [42].

### 2.3. Clinical Characteristics of PCa Cases

The Gleason score was collected from the medical records of cases, and we regrouped as: ISUP 1–2 and ISUP 3–5 [43,44]. Additionally, according to the staging of PCa, we classified cases as localized, locally advanced, or metastatic PCa [45].

### 2.4. Statistical Analysis

To describe the characteristics of PCa cases and controls, the mean and standard deviation of the continuous quantitative variables and the percentages for the categorical variables were calculated. Chi squared tests were used to assess the level of significance of the differences observed in the categorical variables. For continuous variables, the Mann–Whitney U test was calculated.

To reduce the complexity of the data, the 134 FFQ food items were categorized into 28 food and beverage groups (Supplementary Table S1). Food and beverage items were grouped according to similarities in their nutrient profiles and similarly to a previous study [22]. To extract nutrient patterns, principal component analysis was applied. The following criteria were used to determine the number of factors: eigenvalue of the factors ≥1, and identification of a breakpoint in the Scree plot [46]. An orthogonal rotation (varimax) was applied to the factor-loading matrix to improve the meaning and interpretation of the original factors. Food and beverages items with an absolute rotated factor loading ≥|0.2| were displayed. We used that factor loading to define the food groups that characterized each dietary pattern. Next, the statistical intercorrelation of variables and the sampling adequacy were tested using Bartlett’s test of sphericity (*p* < 0.001) and the Kaiser–Mayer–Olkin value (0.70). Dietary patterns were labeled according to the food groups loaded on each retained factor. Each dietary pattern factor score was categorized into tertiles based on the control group cutoff points.

Multivariable logistic regression models were used to estimate odds ratios (OR) and 95% confidence intervals (95% CI) for the association between the dietary patterns and PCa. The first tertile was used as the reference category (minimum adherence to the dietary pattern). To control for potential confounding factors, models included the following adjustment variables: age, educational level, first-degree family history of PCa, BMI, and energy intake. We used the information from previous studies to identify a priori potential confounders. In addition, analyses were stratified according to the aggressiveness and extension of the tumor.

All statistical tests were two-sided, and statistical significance was set at *p* < 0.05. Statistical analyses were performed using the statistical program Stata v.15 (Stata Corp., 2017, College Station, TX, USA).

## 3. Results

### 3.1. Study Population

The main characteristics of the cases and controls are shown in Table 1. Differences were only observed in age, family history of PCa, and energy intake. Thus, PCa cases were slightly older, more frequently had a family history of PCa, and had higher energy intakes than the controls (*p*-value < 0.05). In relation to tumor aggressiveness of PCa cases, three-quarters (75.2%) had a PCa with an ISUP 1 or 2.

### 3.2. Identification of Dietary Patterns

Table 2 shows the rotated factor-loading matrix for the three retained factors. The eigenvalues for those three components were 2.92, 1.80, and 1.61, and the proportion of the variance explained by each one was 10.4%, 6.4%, and 5.7%, respectively, for a total of 22.6% of the variance in the diet accounted for in the study population. The three retained components were identified as distinct dietary patterns, labeled: (i) “Mediterranean”, characterized by high consumption of leafy, fruiting, root, and other vegetables, as well as fruits, white and oily fish, legumes, potatoes, nuts and eggs; (ii) “Western”, involving high consumption of red meat, processed meat, convenience food, sauces, alcohol, high-fat dairy products, and refined cereals; and (iii) “Unhealthy”, characterized by high intake of convenience food, sauces, sugary products, sweets, and others edible fats.

### 3.3. Association between Dietary Patterns and PCa

Table 3 shows the association between dietary patterns and PCa. A dose–response relationship was observed between the Unhealthy pattern and PCa (*p*-trend = 0.035). Those subjects with high adherence (T3) presented the highest odds of PCa, OR_T3vsT1_ = 1.52 (95% CI 1.02–2.27). However, we could not observe a clear association between the Western pattern (OR_T3vsT1_ = 1.25 (95% CI 0.83–1.87)) or the Mediterranean pattern (OR_T3vsT1_ = 0.90 (95% CI 0.63–1.29)) and PCa.

The association between dietary patterns and PCa according to tumor aggressiveness (ISUP) and extension is shown in Table 4. A dose–response relationship was observed between an Unhealthy pattern and ISUP 1–2 tumors (*p*-trend = 0.018); however, no relationship was observed for cases with ISUP 3–5. High adherence (T3) to the Unhealthy pattern was associated with an increase of 67% in odds of ISUP 1–2 tumors compared with subjects with low adherence (T1), OR _T3vsT1_ = 1.67 (95% CI 1.09–2.57). Similarly, these increased odds were observed for localized PCa cases, OR _T3vsT1_ = 1.56 (95% CI 1.03–2.36). For the Mediterranean and Western patterns, a clear trend could not be established regardless of tumor aggressiveness or tumor extension.

## 4. Discussion

Briefly, a positive association was found between high adherence to an Unhealthy pattern (T3) diet and PCa, especially for tumors with an ISUP 1–2 and localized PCa. Although the Mediterranean diet could be inversely associated with the odds of PCa, and the Western diet could increase the odds of this tumor, we were unable to establish a clear relationship between these patterns and PCa for either aggressiveness or extension.

Diet has been studied from multiple approaches. Considering the possible synergies between nutrients and food, it is necessary to consider diet from a global perspective, and principal component analysis is the most-used method to analyze this [17,19,20]. Using this method in the CAPLIFE study, we identified three dietary patterns: (i) “Mediterranean”, (ii) “Western”, and (iii) “Unhealthy”. These dietary patterns can explain 22.6% of the variance, with the Mediterranean pattern explaining the highest percentage of variance in food intake (10.4%), followed by a Western pattern (6.4%), and lastly an Unhealthy pattern (5.7%). These data are very similar to those obtained in previous epidemiological studies that used food groups or foods to identify dietary patterns, where the variance ranged between 11 and 38% [21,25,26,28,29,33,47,48,49].

Our results suggest the existence of a positive association between an “Unhealthy pattern” and PCa. However, comparison with previous studies is very complex, given that: (i) dietary patterns were constructed a posteriori, and, therefore, are dependent on the study population; and (ii) the food and beverage groups included differed between studies. Considering these limitations, our findings are in line with two case-control studies [21,24]. Trudeau et al., in a case-control study carried out in Canada, identified a pattern called “Sweets and Beverages”, characterized by high intakes of pasta, pizza, cookies, chips, chocolate, and carbonated soft drinks, which was associated with increased odds of PCa (OR_Q4vsQ1_ = 1.35 (95% CI 1.10–1.66)) [21]. Similarly, Bagheri identified an “Unhealthy” pattern associated with increased odds of having PCa (OR_T3vsT1_ = 3.4 (95% CI 1.09–10.32)) [24]. However, in our study, this pattern included foods such as red and processed meat or high-fat dairy with an absolute rotated factor loading less than |0.2| [24]. This positive association could be in part because the “Unhealthy pattern” is directly associated with higher energy intake, which may increase the risk of PCa through the production of growth and angiogenesis factors [50,51]. On the other hand, the Western pattern was not associated with PCa, although slightly increased odds were observed. For this pattern, results are contradictory. Neither the MCC-Spain study nor two Canadian case-control studies found an association between a Western pattern and PCa, in line with our results [21,22,49]. However, these results contrast with Australian and Iranian case-control studies and a Japanese cohort study, which propose an association of risk for those men with high adherence to the Western pattern [25,26,27]. A possible explanation could be the inclusion in the Western pattern of sweets, desserts, and carbonated drinks, groups of foods with low rotated factor loadings in the CAPLIFE study and in two of the three case-control studies mentioned above [21,22]. These foods have a high glycemic index and are associated with hyperinsulinemia, which has been associated with an increased risk of cancer [52].

We could not establish a clear association regarding the Mediterranean pattern, although a protective association seems to be observed for PCa. These results align with an Iranian case-control study and the Melbourne Collaborative Cohort Study (MCCS) [27,34]. However, other studies identified a protective association for patterns similar to what is called a healthy or Mediterranean pattern [21,22,28]. The CAPLIFE study was carried out in the south of Spain, with a study population with a typical Mediterranean pattern. This fact could have made it difficult to detect a protective association. Along with this, the inflammatory power of the diet is another aspect to consider when evaluating the dietary pattern [53].

Although many studies have analyzed the role of dietary patterns and PCa, just a few considered tumor aggressiveness in this association [21,22,25,26,33,34]. When we stratified by aggressiveness, a positive association was observed between the unhealthy pattern and cases with ISUP 1–2 and localized tumors. In this sense, MCC-Spain proposes that adherence to a Mediterranean pattern would protect against Gleason > 6 and cT2b-T4 tumors [22]. On the other hand, Ambrosini et al. propose that subjects who adhere to a Western pattern have a higher risk of aggressive PCa (Gleason ≥ 7) [25], while Jackson et al. suggest that those who adhere to a Carbohydrate pattern have a higher risk of low-aggressive PCa (Gleason < 7) [33]. However, these results are hardly comparable to ours due to the different classification of tumor aggressiveness and extension. In relation to tumor aggressiveness, we have used the ISUP classification, which does not consider all Gleason score 7 tumors in the same category: while a Gleason score 3 + 4 is an ISUP 2, a Gleason score 4 + 3 is an ISUP 3.

Among the limitations of this study, labeling of the patterns is mainly subjective and derived from the authors’ criteria. Besides, resulting patterns in posteriori methodologies are specific to the population from which they emerge, which leads to difficulty comparing with other studies. Although, as mentioned above, a posteriori methods are preferable, they do not allow comparability between studies. Along with this, we used terciles constructed from the cutoff points of the control group, so the categories of the patterns also depend on the study population. Although we cannot rule out recall bias, a validated questionnaire for dietary information collection was used to try to minimize it. We consider that if there were recall bias, it would have affected cases and controls in the same way, thus being a nondifferential bias. Moreover, although the FFQ refers to the previous year, dietary habits have been shown to remain fairly stable over time [54].

Our study also has several advantages, and the following should be highlighted: (i) inclusion of a representative sample of incident PCa cases. It should be noted the sample size is larger than that of most previous studies [24,25,27,28,29,33,49,55,56]; (ii) the study of information of multiple food intakes summarized in a unique exposition measurement through principal component analysis constitutes a methodological advantage as it addresses the problem of multicollinearity and simplifies the interpretation of results; (iii) the use of a semi-quantitative FFQ for the evaluation of diet. The FFQ is a validated questionnaire that includes regional products and is recognized as a standard method for assessing habitual dietary intake [38]. Additionally, a person’s diet tends to remain stable over time. In addition to collecting the diet the year prior to diagnosis, PCa risk would not be affected by a possible change in dietary habits after diagnosis [54]; (iv) we have used the ISUP grade grouping to measure aggressiveness—a categorization recommended by the last Consensus Conference on Gleason Grading of Prostatic Carcinoma [44]—and staging of PCa for tumor extension; and (v) most CAPLIFE study participants had detailed information on diet (91.0% of the cases and 91.4% of the controls).

## 5. Conclusions

In conclusion, we observed that men with high adherence to an Unhealthy pattern diet had higher odds of PCa, especially for cases with a ISUP 1–2 and localized PCa. Although the Mediterranean diet could decrease the odds of PCa and the Western diet could increase the odds of this tumor, we were unable to establish a clear relationship between these patterns and PCa, nor when considering tumor aggressiveness and extension. According to these results, diet could be associated with PCa; therefore, future recommendations should focus on avoiding unhealthy dietary patterns.

## Figures and Tables

**Table 1:** Characteristics of PCa cases and controls in the CAPLIFE study.

	Controls *n* = 393	PCa Cases *n* = 428	*p*-Value
Age (years), median (IQR)	66.5 (61.3–72.3)	68.5 (62.8–73.9)	0.005
Education, *n* (%)			0.377
Primary	109 (27.8)	124 (28.9)	
Secondary	199 (50.6)	228 (53.3)	
University	85 (21.6)	76 (17.8)	
Employment status, *n* (%)			0.602
Retired	264 (67.1)	298 (69.7)	
Currently working	103 (26.2)	99 (23.1)	
Unemployed	26 (6.6)	30 (7.0)	
Missing	-	1 (0.2)	
Marital status, *n* (%)			0.760
Married	330 (83.9)	356 (83.2)	
Not married	63 (16.1)	72 (16.8)	
Comorbidities, *n* (%)			0.740
0–2	364 (92.9)	399 (93.4)	
≥3	28 (7.1)	28 (6.6)	
Missing	1	1	
Smoking status, *n* (%)			0.853
Never smoker	104 (26.4)	112 (26.2)	
Former smoker	217 (55.2)	231 (53.9)	
Current smoker	72 (18.4)	85 (19.9)	
BMI, *n* (%)			0.758
Normal weight	72 (18.4)	87 (20.3)	
Overweight	207 (52.6)	219 (51.2)	
Obesity	114 (29.0)	121 (28.3)	
Missing	-	1 (0.2)	
Physical activity, *n* (%)			0.158
High	133 (33.8)	166 (38.7)	
Moderate	201 (51.2)	214 (50.0)	
Low	59 (15.0)	48 (11.3)	
Sedentary behavior (h/day), median (IQR)	7.0 (5.0–9.0)	7.0 (5.0–10.0)	0.268
Energy intake (Kcal/day), median (IQR)	2290.3 (1943.1–2809.4)	2428.9 (2057.5–2918.9)	0.021
Alcohol consumption (g/day), median (IQR)	7.3 (1.4–15.9)	8.0 (1.5–18.5)	0.349
First-degree family history of PCa, *n* (%)			<0.001
No	350 (89.1)	333 (77.8)	
Yes	43 (10.9)	94 (22.0)	
Missing	-	1 (0.2)	
ISUP grade *, *n* (%)			-
1–2	-	321 (75.2)	
3–5	-	106 (24.8)	
Staging of PCa, *n* (%)			
Localized	-	369 (86.2)	
Locally advanced	-	35 (8.2)	
Metastatic	-	24 (5.6)	

BMI, body mass index; IQR, interquartile range (percentile 25-percentile 75). * One subject could not be categorized using ISUP classification as it was a neuroendocrine carcinoma. Note: missing data are not included for comparing cases and controls.

**Table 2:** Rotated factor loadings and explained variances for 3 main dietary patterns identified by principal component analysis.

Food and Beverage Groups	Mediterranean Pattern	Western Pattern	Unhealthy Pattern
High-fat dairy	-	0.37	-
Eggs	0.38	-	-
Red meat	-	0.71	-
Processed meat	-	0.72	
White fish	0.30	-	-
Oily fish	0.27	-	-
Leafy vegetables	0.75	-	-
Fruiting vegetables	0.79	-	-
Root vegetables	0.74	-	-
Other vegetables	0.68	−0.27	-
Legumes	0.21	-	-
Potatoes	0.30	−0.38	-
Fruits	0.35	-	-
Nuts	0.21	-	-
Refined cereals	-	0.22	-
Other edible fats	-	-	0.69
Sweets	-	-	0.62
Sugary	-	-	0.65
Convenience food and sauces	-	0.40	0.42
Alcohol	-	0.36	-
Proportion of variance explained (%)	10.4	6.4	5.7
Cumulative variance explained (%)	10.4	16.9	22.6

Food and beverage groups with loadings <|0.2| are not shown.

**Table 3:** Association between dietary patterns and PCa in the CAPLIFE study.

Dietary Pattern	Controls/PCa Cases	aOR (95% CI)
Mediterranean pattern		
Tertile 1	131/136	Ref.
Tertile 2	131/140	0.92 (0.65–1.31)
Tertile 3	131/152	0.90 (0.63–1.29)
p-trend		0.578
Western pattern		
Tertile 1	131/124	Ref.
Tertile 2	131/157	1.33 (0.93–1.90)
Tertile 3	131/147	1.25 (0.83–1.87)
p-trend		0.289
Unhealthy pattern		
Tertile 1	131/116	Ref.
Tertile 2	131/133	1.11 (0.77–1.60)
Tertile 3	131/179	1.52 (1.02–2.27)
p-trend		0.035

aOR: Odds ratio adjusted for age, educational level, first-degree family history of PCa, BMI, and energy intake.

**Table 4:** Association between dietary patterns and PCa according to tumor aggressiveness (ISUP) and extension in the CAPLIFE study.

Dietary Pattern	PCa Cases (*n*)	aOR (95% CI)	PCa Cases (*n*)	aOR (95% CI)	PCa Cases (*n*)	aOR (95% CI)	PCa Cases (*n*)	aOR (95% CI)
	ISUP 1–2		ISUP 3–5		Localized PCa		Locally Advanced and/or Metastatic PCa	
Mediterranean pattern								
Tertile 1	102	Ref.	33	Ref.	115	Ref.	21	Ref.
Tertile 2	105	0.94 (0.64–1.37)	35	0.92 (0.53–1.62)	122	0.96 (0.67–1.38)	18	0.70 (0.35–1.40)
Tertile 3	114	0.94 (0.63–1.39)	38	0.86 (0.49–1.51)	132	0.94 (0.65–1.37)	20	0.69 (0.34–1.38)
p-trend		0.746		0.605		0.754		0.293
Western pattern								
Tertile 1	97	Ref.	27	Ref.	104	Ref.	29	Ref.
Tertile 2	113	1.23 (0.84–1.82)	45	1.54 (0.86–2.75)	135	1.37 (0.94–1.99)	42	1.10 (0.55–2.20)
Tertile 3	113	1.26 (0.81–1.94)	34	1.19 (0.60–2.34)	130	1.30 (0.86–1.98)	33	0.93 (0.40–2.15)
p-trend		0.302		0.642		0.223		0.881
Unhealthy pattern								
Tertile 1	82	Ref.	34	Ref.	98	Ref.	18	Ref.
Tertile 2	103	1.22 (0.82–1.81)	29	0.81 (0.45–1.46)	115	1.11 (0.76–1.63)	18	1.01 (0.49–2.09)
Tertile 3	136	1.67 (1.09–2.57)	43	1.16 (0.63–2.13)	156	1.56 (1.03–2.36)	23	1.31 (0.61–2.84)
p-trend		0.018		0.638		0.032		0.488

aOR: Odds ratio adjusted for age, educational level, first-degree family history of PCa, BMI, and energy intake.

## Data Availability

Not applicable.

## Supplementary Materials

The following supporting information can be downloaded at: https://www.mdpi.com/article/10.3390/cancers14143475/s1, Table S1: Food and beverage groups and items included in each of them.
