# Supplementary material for: Dietary Patterns and Prostate Cancer: CAPLIFE Study
Source: Cancers (Basel). 2022 Jul 17;14(14):3475. doi: 10.3390/cancers14143475 (PMC9316982; doi:10.3390/cancers14143475)
Supplement: Supplementary file 1 [file cancers-14-03475-s001.zip › cancers-1809392-supplementary.pdf]

Supplementary Table S1. Food and beverage groups and items included in each of them.

Food and beverage groups	Food and beverage items
**High-fat dairy**	Whole milk, condensed milk, cream or milk cream, milk shakes, whole yogurt, petit suisse, portioned or creamy cheese, other cheeses: cured, semi-cured (Manchego, Bola, Emmental...), custard, flan, pudding, ice cream
**Low-fat dairy**	Semi-skimmed milk, skimmed milk, low-fat yogurt, cottage cheese or curd, white or fresh cheese (Burgos, goat...)
**Eggs**	Eggs
**White meat**	Turkey or chicken with skin, turkey or chicken without skin, rabbit or hare
**Red meat**	Veal or beef, pork, lamb, liver (veal, pork, chicken), other viscera (brains, gizzards, kidneys), serrano ham or shoulder, hamburger, meatballs
**Processed meat**	York ham, cooked ham, processed meats (sausage, chorizo, black pudding, mortadella, sausages, botifarra, sobrassada), pâtés, foie gras, bacon, bacon, pancetta
**White fish**	Grouper, sole, sea bream, hake, whiting
**Oily fish**	Sardines, tuna, bonito, mackerel, salmon, natural canned fish and shellfish (sardines, anchovies, bonito, tuna), canned fish and shellfish with oil (sardines, anchovies, bonito, tuna)
**Seafood/Shellfish**	Oysters, clams, mussels and the like, squid, octopus, baby squid, cuttlefish (cuttlefish), crustaceans (prawns, prawns, crayfish, etc.)
**Leafy vegetables**	Swiss chard, spinach, lettuce, endives, escarole
**Fruiting vegetables**	Tomatoes, aubergines, courgettes, cucumbers, peppers
**Root vegetables**	Carrot, pumpkin
**Other vegetables**	Cabbage, cauliflower, broccoli, green beans, asparagus, Andalusian gazpacho, other vegetables (artichoke, leek, thistle, celery), onions, garlic, mushrooms, chanterelles, mushrooms, parsley, thyme, bay leaf, oregano
**Legumes**	Lentils, beans (pinto, white or black), chickpeas, peas, broad beans
**Potatoes**	Backed or cooked potatoes
**Fruits**	Orange, grapefruit, tangerines, banana, apple or pear, strawberries, cherries, picotas, plums, peaches, apricots, nectarine, watermelon, melon, kiwi, grapes, olives, fruits in syrup or in its juice, dates, dried figs, raisins, prunes
**Nuts**	Almonds, peanuts, hazelnuts, pistachios, pine nuts, walnuts
**Refined cereals**	White bread, sliced bread, breakfast cereals, white rice, pasta (noodles, macaroni, spaghetti, others)
**Whole grains**	Black or whole-grain bread, whole-grain cereals (muesli, oat flakes, all-bran)
**Olives and vegetable oil**	Olives, olive oil, extra virgin olive oil, pomace olive oil, corn oil, sunflower oil, soybean oil, mixture of the above
**Other edible fats**	Margarine, butter, lard
**Sweets**	Biscuits, wholemeal or fiber biscuits, chocolate biscuits, homemade pastries and biscuits, croissants, Mallorcan pastry, tea cakes, other commercial industrial pastries, donuts, muffins, cakes, chocolates and chocolates, cocoa powder, soluble cocoa, nougat, mantecados, marzipan, churros, batons and the like
**Sugary**	Fruits in syrup, jam, sugar, honey
**Juices**	Natural orange juice, natural other fruit juice, bottled or canned fruit juice

**Caloric drinks**	Carbonated drinks with sugar (cola drinks, lemonades, tonics, etc.), low-calorie carbonated drinks, light drinks
**Convenience food and sauces**	Commercial French fries, homemade French fries, pizza, croquettes, fritters, dumplings, precooked, soups and cream packets, commercial mayonnaise, fried tomato sauce, ketchup, spicy (tabasco, pepper, paprika), salt, snacks other than chips (worms, popcorn, corn, etc.)
**Alcohol**	Must, rosé wine, muscatel wine, young red wine, aged red wine, white wine, cava, beer, liqueurs, aniseed or aniseed, distillates (whiskey, vodka, gin, cognac)
**Calorie free drinks**	Tea, decaffeinated coffee, coffee
